# Cyanidiophyceae (Rhodophyta) Tolerance to Precious Metals: Metabolic Response to Palladium and Gold

**DOI:** 10.3390/plants10112367

**Published:** 2021-11-03

**Authors:** Maria Sirakov, Maria Palmieri, Manuela Iovinella, Seth J. Davis, Milena Petriccione, Maria Rosa di Cicco, Mario De Stefano, Claudia Ciniglia

**Affiliations:** 1 Department of Biology and Evolution of Marine Organisms, Stazione Zoologica Anton Dohrn, Villa Comunale, 80121 Naples, Italy; maria.sirakov@szn.it; 2 Department of Environmental, Biological and Pharmaceutical Sciences and Technologies, Università Degli Studi di Caserta “L. Vanvitelli”, Via Vivaldi 43, 81100 Caserta, Italy; maria.palmieri@unicampania.it (M.P.); mariarosa.dicicco@unicampania.it (M.R.d.C.); mario.destefano@unicampania.it (M.D.S.); claudia.ciniglia@unicampania.it (C.C.); 3 Department of Biology, University of York, York YO10 5DD, UK; seth.davis@york.ac.uk; 4 State Key Laboratory of Crop Stress Biology, School of Life Sciences, Henan University, Kaifeng 475004, China; 5 Consiglio per la Ricerca in Agricoltura e L’analisi Dell’economia Agraria (CREA)-Unità di Ricerca per la Frutticoltura, Via Torrino 3, 81100 Caserta, Italy; milena.petriccione@crea.gov.it

**Keywords:** metal tolerance, *Galdieria sulphuraria*, precious metals, metabolic response

## Abstract

Polyextremophilic red algae, which belong to the class *Cyanidiophyceae*, are adapted to live in geothermal and volcanic sites. These sites often have very high concentrations of heavy and precious metals. In this study, we assessed the capacity of three strains of *Galdieria* (*G. maxima*, *G. sulphuraria*, and *G. phlegrea*) and one strain of *Cyanidium* *caldarium* to tolerate different concentrations of precious metals, such as palladium (Cl_4_K_2_Pd) and gold (AuCl_4_K) by monitoring algal growths in cultures exposed to metals, and we investigated the algae potential oxidative stress induced by the metals. This work provides further understanding of metals responses in the *Cyanidiophyceae*, as this taxonomic class is developed as a biological refinement tool.

## 1. Introduction

Recently, there has been a remarkable and growing demand for recovering elements and energetic resources from waste streams [1]. In particular, high levels of concern have been directed towards precious metals and rare earth elements (REEs) due to their extensive use in superconductors, catalysts, and electronics industries. Conversely, their discharge into the environment and the suitability of recycling from e-waste are relevant topics because of their environmental and health hazards, in addition to their economic value. These issues have become evident to governments and to electronics industries that are increasingly prone to develop new methods to remove REEs from the environment and to possibly recycle them back into a “closed-loop economy” production cycle [2,3,4,5,6], while simultaneously achieving energy optimization goals [7,8]. Recently, biological methods have been developed to ensure the recovery of small quantities of these metals from wastewater systems [5], using mainly bacteria [9,10,11,12] or plants known for their ability to immobilize heavy metals in the cell wall and to compartmentalize them in vacuoles [13]. Interestingly, polyextremophilic algae have intrinsic properties that make them capable of selective removal and concentration of metals, thanks to their adaptation to live in geothermal and volcanic sites [14,15,16,17]. Geothermal fluids leach out of hot volcanic rocks and are enriched by enormous amounts of minerals and metals, including lithium, sulphur, boric acid, and precious metals such as gold, platinum, palladium, and silver [18].

Cyanidiophyceae, a class of unicellular red algae, thrive in extreme conditions, very low pH (0.0–3.0), and high temperatures (37–55 °C), and colonize acid and hydrothermal sites, in addition to rocks and muddy soil around hot ponds [19]. They are divided into three genera: *Cyanidioschyzon*, *Cyanidium,* and *Galdieria*, which differ in size, cellular shape, and growth conditions. *Cyanidioschyzon merolae*, the only species belonging to the *Cyanidioschyzon* genus, differs from the other two taxa due to the lack of a cell wall and division by binary fission [20]. *Cyanidioschyzon*, *Cyanidium,* and *Galdieria* can grow on ammonia as well as nitrate. The *Cyanidioschyzon* and *Cyanidium* species are obligatory autotrophs, while the *Galdieria* species can grow auto-, mixo-, and heterotrophically and tolerate high concentrations of salts [21]. This makes *Galdieria* particularly suitable for biotechnological applications [14]. The ability of *Galdieria sulphuraria* to recover REEs has already been assessed [5,22] and confirmed by an approved patent [23]. In this study, we focused an in depth, comparative study on the ability of different *Galdieria* species (*Galdieria maxima*, *Galdieria sulphuraria*, and *Galdieria phlegrea*) and *Cyanidium caldarium* to tolerate different concentrations of precious metals (palladium Cl_4_K_2_Pd and gold AuCl_4_K). We also investigated the metabolic response and oxidative stress induced by these metals by monitoring superoxide dismutase (SOD), catalase (CAT), and ascorbate peroxidase (APX) activities.

## 2. Results

Polyextremophilic microalgae, such as Cyanidiophyceae, have a high intrinsic capacity to uptake metals, involving active and passive mechanisms [5]. Heavy, rare, or precious metals can influence algae physiology in various ways, likely inhibiting different physiological processes. To evaluate the suitability of Cyanidiophyceae, other than *G. sulphuraria,* for biotechnological application to effectively recover precious metals, we tested their tolerance to Cl_4_K_2_Pd and AuCl_4_K by monitoring the growth and metabolic response of four different taxa, which were exposed to each of these metals at a concentration in the range 1–10 g/L. As discussed in depth in Section 4, the growth was evaluated after 4 days (96 h) from a single metal exposure. The results were expressed in the form of maximum growth rate (MGR).

**Figure 1:**
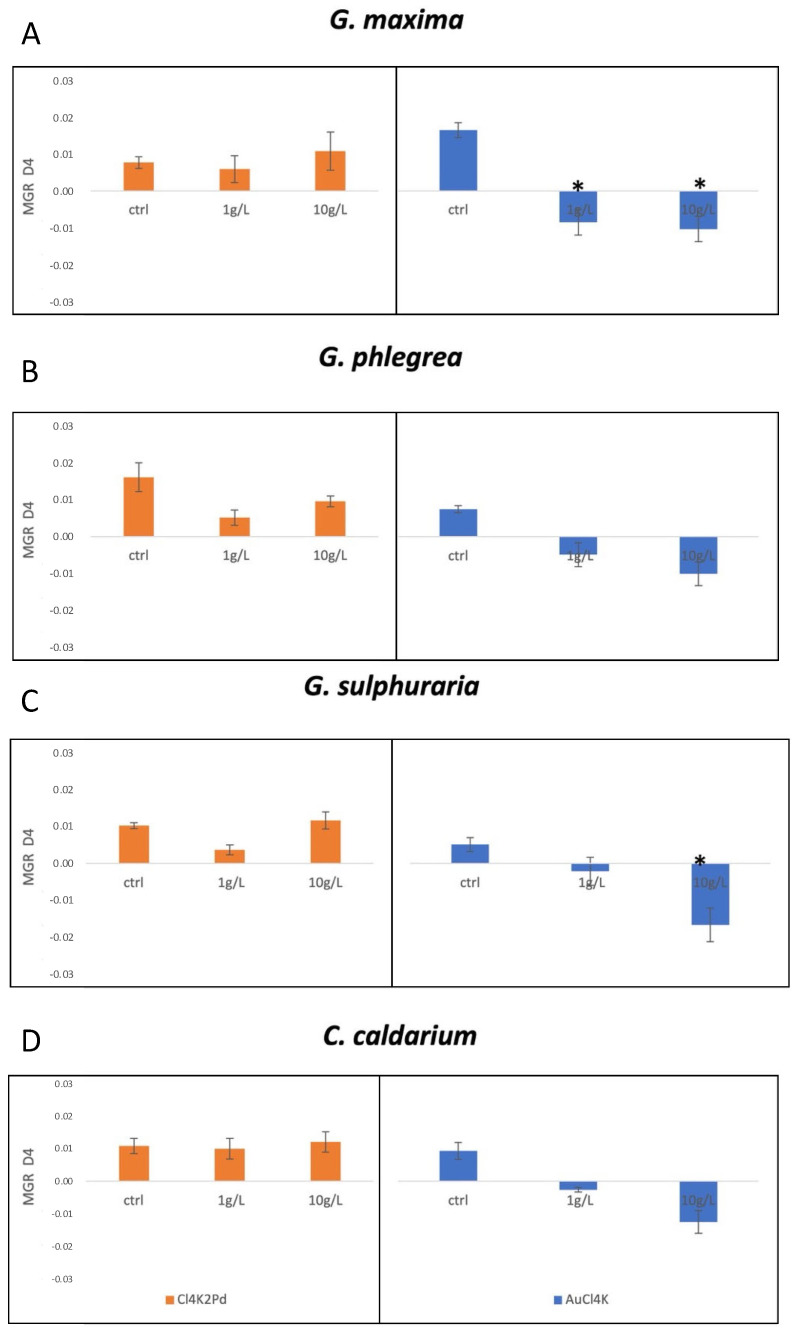
Evaluation of metal tolerance through MGR monitoring after 4 days (96 h). Maximum growth rate in the presence of different concentrations of palladium (Cl_4_K_2_Pd, orange, left panel) and gold (AuCl_4_K, blue, right panel), for the species: (**A**) *G.* *maxima;* (**B**) *G.* *phlegrea;* (**C**) *G. sulphuraria;* (**D**) *C.* *caldarium*. Error bars represent standard deviation of three replicates. (*) = *p*-value ≤ 0.000000001 calculated by *T*-test.

As shown in Figure 1, the presence of AuCl_4_K significantly reduced cellular duplication in *G. maxima* at all the concentrations tested; Cl_4_K_2_Pd did not negatively affect cell growth, and no statistical difference was recorded between MGR in the control and tests (Figure 1A). Regarding *G. phlegrea*, both metals induced a trend of reduction in growth rate at both concentrations (Figure 1B) and *vice versa*, in *G. sulphuraria*, AuCl_4_K reduced cell growth at the maximum concentration, while MGR appeared to be not affected by Cl_4_K_2_Pd, as shown by the MGR values at 10 g/L, as compared with the control. A decrease in growth rate was recorded at a lower concentration (1 g/L); presumably, higher levels of palladium was beneficial for the growth of this strain. Perhaps this improvement of cell duplication can be interpreted as a defense of the algal strain. Finally, *C. caldarium* showed a high tolerance to Cl_4_K_2_Pd, whereas AuCl_4_K significantly inhibited cell duplication as the metal concentration increased (Figure 1D). The highest concentration of palladium (10 g/L) improved the growth, and in *G. maxima* and *G. sulphuraria*, the MGR values outperformed the controls.

**Figure 2:**
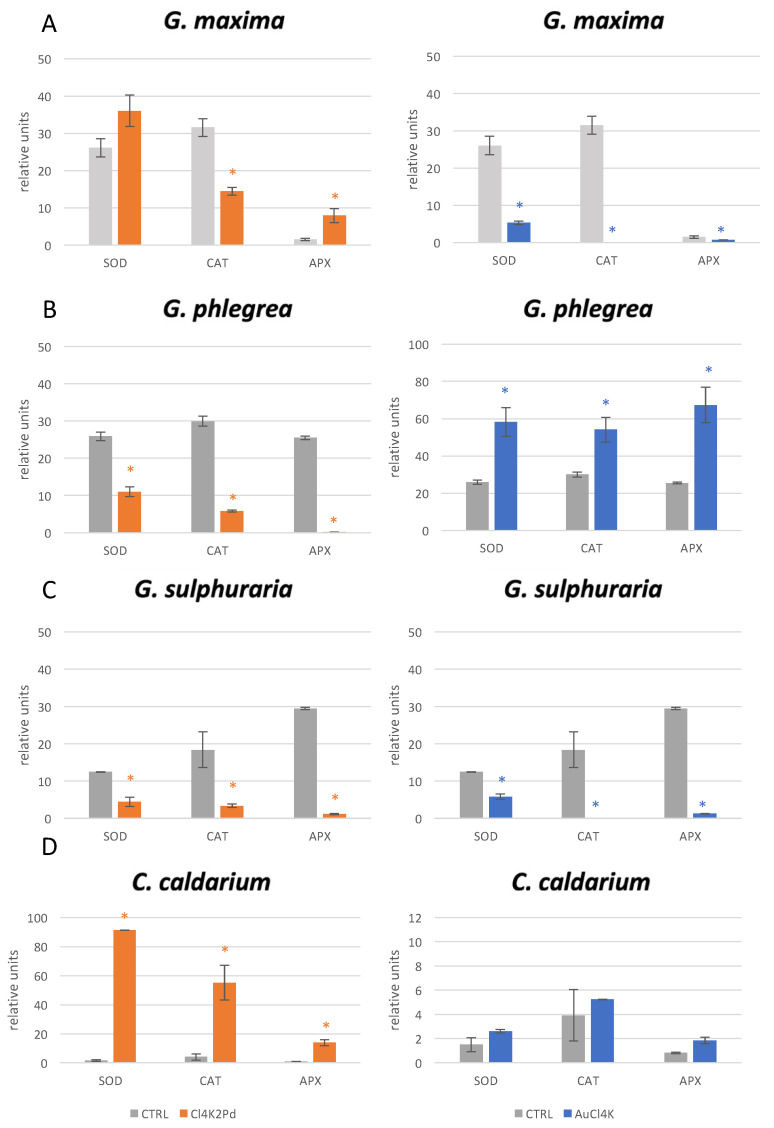
Evaluation of enzymatic activities after metals exposure. Relative units represent enzymatic activity as units/g of dry weight (SOD), enzymatic activity as nmol H_2_O_2_/g of fresh weight (CAT), and enzymatic activity as μmol ascorbate/g of fresh weight (APX). Enzymatic activities were monitored in: (**A**) *G.* *maxima;* (**B**) *G. phlegrea;* (**C**) *G. sulphuraria;* (**D**) *C. caldarium*, treated with 1 g/L of palladium (Cl_4_K_2_Pd, orange bars) and gold (AuCl_4_K, blue bars) after 96 h. Mean (± SD) was calculated from three replicates. (*) = *p*-value ≤ 0.05 calculated by *T*-test.

We next evaluated the ROS scavenging activities of SOD, CAT, and APX in all the algae tested in the presence of Cl_4_K_2_Pd and AuCl_4_K. This as performed at a concentration of 1 g/L, after an incubation period of 24 h. The reason for this choice was because the antioxidant activity was considered to be a measure of cell effectiveness in response to the impact of metals, increasing their tolerance as a protective mechanism necessary to remove ROS before they could damage sensitive parts of the cellular machinery. In particular, SOD, which catalyses the dismutation of O_2_^−^ (singlet oxygen) to O_2_ and H_2_O_2_, was defined as the first cellular defence against ROS production. Meanwhile, CAT catalyses the production of H_2_O from the degradation of H_2_O_2_ and ROOH. Finally, APX reduces H_2_O_2_ to H_2_O using the ascorbate as an electron donor. The strain-/metal-specific metabolic responses were quite diverse, as shown in Figure 2. Indeed, APX activity significantly increased only in *G. maxima* in response to Cl_4_K_2_Pd, while in the presence of AuCl_4_K, all the enzymatic activities appeared to be reduced (Figure 2A). Concerning the other strains, in *G. phlegrea*, all the tested enzymatic activities decreased in the presence of Cl_4_K_2_Pd and increased in the presence of AuCl_4_K (Figure 2B); in *G. sulphuraria*, both metals induced a decrease in enzymatic activity (Figure 2C). Finally, we observed a significant increase in all the enzymatic activities in *C. caldarium* in the presence of Cl_4_K_2_Pd, but not in the presence of AuCl_4_K (Figure 2D).

## 3. Discussion

A significant increase of enzymatic activity in response to metals addition, as compared with the control, suggests a high scavenging activity of the singlet oxygen in hydrogen peroxide. This provides evident tolerance of these algae to the metal under examination. Increases in the activities of both antioxidant enzymes are necessary to reduce the concentrations of both singlet oxygen and hydrogen peroxide, minimizing the risks. In general, modulation of antioxidant enzymes is an essential adaptive response to counteract adverse conditions; in fact, maintaining a high antioxidant capacity in the cells is correlated with increased tolerance against different types of environmental stress [24].

Our results indicate that precious metals can be tolerated by all the strains tested, although there is, clearly, a higher tolerance to Cl_4_K_2_Pd vs. AuCl_4_K when considering growth rates. The comparison of growth rates in the presence of different concentrations of the metals showed that *G. phlegrea* appears to be more affected by the presence of either metals, as it showed a decrease in both growth and metabolic responses. The contribution to the oxidative equilibrium of the examined extremophile microalgae and the induction of antioxidant enzymes could result from the adaptation of the cell to the development of intracellular ROS. However, there was no clear correlation between any enzymatic activity and the better performing growth of the other three strains tested.

Although metals application generally induces inhibition of microalgal growth, several reports have also suggested their positive roles. It is known that metals at small concentrations are useful for microalgal metabolism since they participate in the synthesis of proteins involved in photosynthesis, nitrogen assimilation, phosphorous acquisition, CO_2_ fixation, and DNA transcription [25]. Algae can develop efficient defense mechanisms to counteract the toxicity and to improve their survival, even at high metal concentrations [26]. One of the defense strategies is the accumulation of the metals, which consists of metal adsorption on the cell surface (biosorption), followed by their entry into the cell protoplast (bioaccumulation). When metals accumulate inside the cell, the algae activate molecular mechanisms as other defense strategies to reduce their toxicity [26]. *G. sulphuraria* can survive in harsh environments rich in heavy, precious and rare-earth metals by detoxifying and transforming them into less toxic derivatives [27]. The defense strategies developed by algae to prevent the toxic effect of some metals represent a good opportunity for biotechnological purposes. A study by Ju et al., (2016) showed that the ability of *G. sulphuraria* to recover both Cl_4_K_2_Pd and AuCl_4_K was inefficient [5]. However, the authors did not test this strain’s tolerance to growth in the presence of these metals. In contrast, we consider that tolerance and growth capacity is an essential parameter to be considered for biotechnological applications, such as metals recovery.

## 4. Materials and Methods

### 4.1. Strain Cultivations

The algal strains used in this study belong to the algal collection of the University of Campania “L. Vanvitelli” derived from the University of Naples (www.acuf.net; accession date 10 August 2021), namely ACUF 3.4.5 (*G. maxima*), ACUF 7.6.21 (*G. phlegrea*), ACUF 9.2.11 (*G. sulphuraria*), and ACUF 626 (*C. caldarium*). All the strains were inoculated in Allen medium containing (NH_4_)_2_SO_4_ as the nitrogen source, at a pH of 1.5 by adding H_2_SO_4_ [28], cultivated at 37 °C, and kept mixed on an orbital shaker under a photon irradiance of 150 μmol photons m^−2^ s^−1^ with a 16/8 h light/dark cycle provided by cool-light fluorescent lamps (Philips TLD30w/55, Philips Lighting, Eindhoven, the Netherlands). Cell densities of the algal cultures were assessed, and the optical density (OD) was recorded at 750 nm with a spectrophotometer (Bausch & Lomb Spectronic 20).

### 4.2. Experimental Procedure

Microalgal cultures at an exponential phase were inoculated into fresh Allen medium enriched with Cl_4_K_2_Pd and AuCl_4_K at concentrations ranging from 1 to 10 g/L. The growth rates were calculated within 96 h, using the spectrophotometric measurements of optical density (OD 550 nm, Bausch & Lomb Spectronic 20, Bausch & Lomb, Milan, Italy), and then were used in the following equation for the maximum growth rate (MGR):

MGR (1/d) = (Ln(Nt) − Ln(N0))/(t − t0)

where Nt is the optical density at the final time, N0 is the optical density at the initial time, t is the final time (days), and t0 is the initial time (days).

All analyses were performed in triplicate.

### 4.3. Enzyme Extraction and Assays

Algal cultures grown in the presence of the minimal dose of palladium and gold (1 g/L) were harvested by centrifugation at 14,000 rpm for 10 min after 96 h of exposure. The algal pellets were washed using KH_2_PO_4_ (0.1 M pH 7.8) followed by centrifugation at 12,000 rpm for 4 min at 4 °C, twice. Proteins were extracted and the sample was homogenized with liquid nitrogen using a mortar and a pestle. The obtained powder was resuspended in 3 mL of Lysis Buffer (0.5 M KH_2_PO_4_, pH 7.8, 2 mM DTT, 1 mM EDTA, 1 mM PMSF, and 1.25 mM PEG) and centrifuged at 14,000 rpm for 20 min at 4 °C. The supernatant was used for measurement after Bradford quantification.

The SOD (EC 1.15.1.1) activity was assayed by the photochemical inhibition nitro blue tetrazolium (NBT) method [6]. The reaction mixture contained 50 mM sodium phosphate buffer (pH 7.8), 13 mM methionine, 75 mM NBT, 0.1 mM EDTA, 30 μL of enzyme extract, and 2 mM riboflavin. The reaction was started by switching on the light (two 15 W fluorescent lamps) for 15 min, and the absorbance was measured at 560 nm. Two samples without the enzymatic extract and illumination were used as the controls. One SOD unit was defined as the amount of enzyme corresponding to 50% inhibition of the NBT reduction. The enzyme activity was expressed as units per 1 mg of protein (U mg^−1^ protein).

The CAT (EC 1.11.1.6) activity was assayed according to Aebi (1984) [29], with minor modifications. The H_2_O_2_ decrease was determined after the reaction of the extract in the presence of 50 mM potassium phosphate buffer (pH 7.0) containing 20 mM H_2_O_2_. The reaction was monitored, measuring the decrease in the absorbance at 240 nm for 100 s. The CAT activity was calculated according to the molar extinction coefficient of H_2_O_2_ (39.4 mM^−1^ cm^−1^) and expressed as nmol H_2_O_2_ min^−1^ mg^−1^ protein.

The APX (EC 1.11.1.1) activity was assayed according to Nakano and Asada (1981) [30]. The ascorbate oxidation was determined using the reaction mixture containing 50 mM potassium phosphate buffer (pH 7.0), 0.1 mM EDTA-Na2, 0.5 mM ascorbic acid, and 100 µL of crude enzyme extract. The reaction started by adding 0.1 mM H_2_O_2_, monitoring the decreasing absorbance at 290 nm for 100 s. The APX activity was calculated according to the molar extinction coefficient of ascorbate (2.8 mM^−1^ cm^−1^), expressed as nmol from H_2_O_2_ min^−1^ mg^−1^ protein.

Each condition for each experimental approach was tested 3 times independently.

## 5. Conclusions

Our observations strongly suggest that strains other than *G. sulphuraria* can be used to recover metals due to their high tolerance to precious and heavy metals. Nevertheless, further studies will be necessary to clarify the biological mechanisms underlying the tolerance capacity of *Cyanidiophyceae* and their strategies to respond to metal toxicity for future biotechnological applications.

## Data Availability

The data presented in this study are available on request from the corresponding author. The data are not publicly available due to privacy restriction.
